# Curcumin Loaded Dendrimers Specifically Reduce Viability of Glioblastoma Cell Lines

**DOI:** 10.3390/molecules26196050

**Published:** 2021-10-06

**Authors:** John Gallien, Bhairavi Srinageshwar, Kellie Gallo, Gretchen Holtgrefe, Sindhuja Koneru, Paulina Sequeiros Otero, Catalina Alvarez Bueno, Jamie Mosher, Alison Roh, D. Stave Kohtz, Douglas Swanson, Ajit Sharma, Gary Dunbar, Julien Rossignol

**Affiliations:** 1 Field Neurosciences Institute Laboratory for Restorative Neurology, Central Michigan University, Mt. Pleasant, MI 48859, USA; galli2j@cmich.edu (J.G.); srina1b@cmich.edu (B.S.); gallo1k@cmich.edu (K.G.); koner1s@cmich.edu (S.K.); otero1p@cmich.edu (P.S.O.); bueno1c@cmich.edu (C.A.B.); dunba1g@cmich.edu (G.D.); 2 Program in Neuroscience, Central Michigan University, Mt. Pleasant, MI 48859, USA; kohtz1d@cmich.edu; 3 College of Medicine, Central Michigan University, Mt. Pleasant, MI 48859, USA; 4 Biochemistry, Cellular and Molecular Biology Program, Central Michigan University, Mt. Pleasant, MI 48859, USA; 5 Department of Chemistry and Biochemistry, Central Michigan University, Mt. Pleasant, MI 48859, USA; holtg1ge@cmich.edu (G.H.); jlmosher23@gmail.com (J.M.); alroh@umich.edu (A.R.); swans1d@cmich.edu (D.S.); sharm1a@cmich.edu (A.S.); 6 Field Neurosciences Institute, St. Mary’s of Michigan, Saginaw, MI 48604, USA; 7 Department of Psychology, Central Michigan University, Mt. Pleasant, MI 48859, USA

**Keywords:** PAMAM dendrimers, curcumin, toxicology, nano-molecule, therapy, glioblastoma, cancer

## Abstract

Glioblastoma (GB) is a deadly and aggressive cancer of the CNS. Even with extensive resection and chemoradiotherapy, patient survival is still only 15 months. To maintain growth and proliferation, cancer cells require a high oxidative state. Curcumin, a well-known anti-inflammatory antioxidant, is a potential candidate for treatment of GB. To facilitate efficient delivery of therapeutic doses of curcumin into cells, we encapsulated the drug in surface-modified polyamidoamine (PAMAM) dendrimers. We studied the *in vitro* effectiveness of a traditional PAMAM dendrimer (100% amine surface, G4 NH_2_), surface-modified dendrimer (10% amine and 90% hydroxyl-G4 90/10-Cys), and curcumin (Cur)-encapsulated dendrimer (G4 90/10-Cys-Cur) on three species of glioblastoma cell lines: mouse-GL261, rat-F98, and human-U87. Using an MTT assay for cell viability, we found that G4 90/10-Cys-Cur reduced viability of all three glioblastoma cell lines compared to non-cancerous control cells. Under similar conditions, unencapsulated curcumin was not effective, while the non-modified dendrimer (G4 NH_2_) caused significant death of both cancerous and normal cells. By harnessing and optimizing the components of PAMAM dendrimers, we are providing a promising new route for delivering cancer therapeutics. Our results with curcumin suggest that antioxidants are good candidates for treating glioblastoma.

## 1. Introduction

Glioblastomas (GB) are among the most common and malignant brain tumors of the central nervous system, impacting more than 10,000 people in the United States each year with a median survival rate of approximately 15 months in patients receiving treatment [1]. Treatment typically includes a combination of radiation, neurosurgery, and chemotherapy with temozolomide (TMZ). However, despite great advances in these treatments, the prognosis for GB patients has remained relatively stable over the last three decades, with less than 5% of GB patients living past 5 years, and no reports of cured patients [2]. Outcomes remain poor, due to resistance to treatment and a lack of other targeted therapies [3]. Furthermore, translational efficacy of therapeutics moving from preclinical to clinical use has been particularly poor for glioblastoma.

Temozolomide is the current gold standard of treatment for GB, typically given concomitantly with radiation [4]. However, in nearly 50% of patients this drug is ineffective, largely due to drug resistance linked to a genetic predisposition and methylation of the MGMT gene (O^6^-methylguanine methyltransferase), and/or lack of a DNA repair pathway in GB cells [5]. Due to the highly resistant, infiltrative, and aggressive nature of this disease, new treatments are essential to improve the prognosis of current and future patients.

Curcumin (diferuloylmethane) is a polyphenol isolated from the rhizome of the C. longa plant that has been shown to have anticancer effects, primarily due to its antioxidant and anti-inflammatory properties [6]. Curcumin has been studied for the treatment of many diseases including Alzheimer’s disease [7], diabetes [8], hepatitis, and a variety of different cancers, including pancreatic cancer, breast cancer and glioblastoma [9,10,11,12]. While initial studies using curcumin *in vitro* and with rodent models *in vivo* showed great promise, human clinical trials repeatedly failed to show the same therapeutic value even at doses as high as 8 g per day [13]. 

There are three major pitfalls to the use of curcumin that prevent its therapeutic actions: poor bioavailability, rapid clearance from the body, and rapid metabolism [14]. Previous attempts to facilitate systemic delivery of curcumin, such as packaging in liposomes, have been largely unsuccessful, due to the poor stability of liposomes and the tendency of their external lipid membrane to conjugate to other lipids in the body [15]. Curcumin alone can cross the blood brain barrier (BBB) in small amounts, but packaging curcumin inside a vehicle such as a liposome has great potential, but their size often prevented them from crossing the intact BBB. While the BBB is disrupted in glioblastoma, the tumor environment and poor vascularization around the tumor prevent even some of the smallest particles from reaching the inner core of a tumor mass [16]. Other novel vehicles are composed of organic compounds which lack solubility in water. Therefore, vehicles that are capable of delivering therapeutics, while still maintaining a size small enough to penetrate the blood brain tumor barrier (BBTB), are needed.

Nano-molecules have been used for almost a century in industrial fields such as construction, agriculture, and carbon-based fuels; more recently, some have been adapted for use in the biomedical sciences [17]. First conceptualized in the late 1970s, hyperbranched “starburst polymers” or “dendrimers” were first synthesized in the early 1980s [18]. Dendrimers differ from other monomers and oligomers in their highly dendritic branching density and functional terminal end groups. Their small size (1–10 nm) and the potential to manipulate and control separate components of a dendrimer, make dendrimers suitable candidates for several biomedical applications, including delivery of genes, encapsulated non-soluble molecules, and drugs conjugated to the surface of dendrimers [19,20]. 

**Figure 1:**
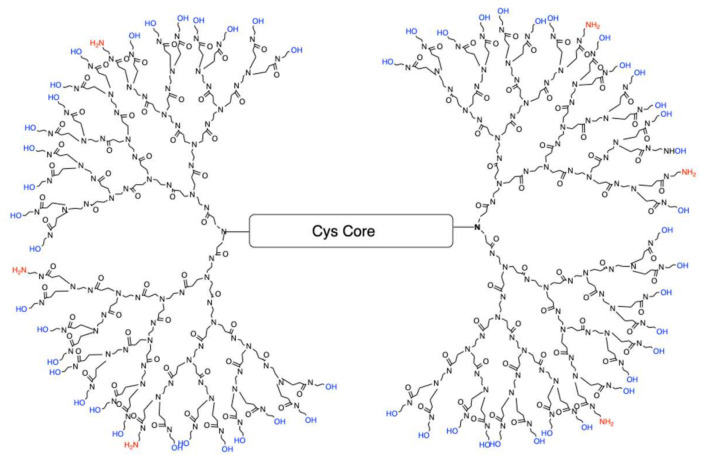
Configuration of G4 90/10-Cys in which 90% of the surface groups are –OH (blue), leaving only 10% as NH_2_ (red). The core is cystamine.

Polyamidoamine (PAMAM) dendrimers are comprised of a core, a branched interior structure, and functional surface groups. Each of these components can be modified to yield dendrimers with a unique phenotype tailored to specific therapeutic actions. Traditional fourth generation PAMAM dendrimer with 100% NH_2_ surface, which exist as protonated positively charged amines (NH_3_^+^) at physiological pH, are highly toxic to cells [21]. Therefore, our lab has developed a reproducible *de novo* method of synthesizing fourth-generation dendrimers (G4) with a modified surface containing fewer positively charged amines resulting in reduced cellular toxicity. One of the current formulations is a G4 dendrimer with 90% of the amine surface replaced with neutral hydroxyl (OH) groups referred to as G4 90/10-Cys (Figure 1).

**Figure 2:**
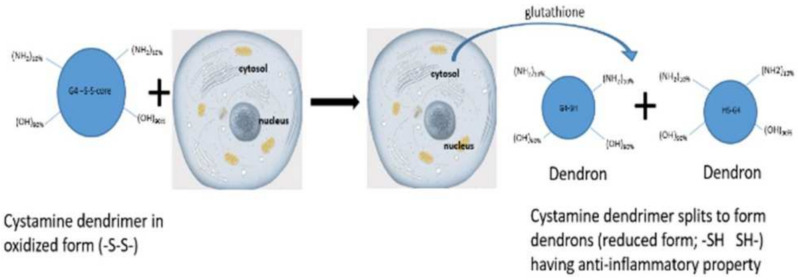
Dendrimer splitting. Upon entering the cell through non-mediated endocytosis, the dendrimer’s core gets reduced from -S-S- to -SH, splitting the dendrimer into two “dendrons”.

The core of G4 90/10 is composed of cystamine, with a disulfide bond holding together the two halves of the dendrimer, or “dendrons.” As shown in Figure 2, upon entering a cell, glutathione or other reducing agents reduce and break the disulfide bond, thus splitting the dendrimer into two dendrons [22], facilitating cargo release (e.g., curcumin) into the interior of the cell.

Recent studies suggest that the polyphenol curcumin, in addition to its many anticancer effects, may also function as a “drug resistance preventer” in certain types of cancers, including glioblastoma [23]. Due to its potential therapeutic value, we created a novel dendrimer/curcumin formulation (G4 90/10-Cys-Cur) for use in this study. The G4 90/10-Cys-Cur has a ratio of 10:1 (*w*/*w*) dendrimer to curcumin. On a mole basis, the ratio is four curcumin per dendrimer. The curcumin-encapsulated dendrimer may be used as a treatment, themselves, or possibly as an adjunctive treatment to those resistant to TMZ or other chemotherapy drugs. 

A common practice in drug development is to investigate the effectiveness of treatments in lower forms of mammals before moving to non-human primates and humans as test subjects. The guidelines for multiple dosing toxicity assessment were developed in 1940, which included multispecies testing as a basic requirement for new drugs, prior to clinical applications [24]. These multispecies preclinical drug testing approaches were further encouraged by study reports about various pharmacological responses in different species to the same drug. Therefore, to account for multispecies variability, we tested our dendrimer treatments on U87-human, F98-rat, and GL261-mouse glioblastoma cell lines, and healthy control cell lines using HEK 293, rat mesenchymal stem cells (MSCs), and mouse-MSCs.

The goal of the study was to develop modified PAMAM dendrimers of different formulations and encapsulating curcumin as potential treatment for glioblastoma in rodent and non-rodent cell lines. Efficacy and specificity of treatment toward cancer cells was assessed using 3-(4,5-dimethylthiazol-2-yl)-2,5-diphenyltetrazolium bromide (MTT) toxicity assays. Although qualitative assessments of cell death included observations of floating cells, accumulation of cell fragments and precipitated material, change in media color, etc., the MTT assay was used to quantify the cell death. The MTT is commonly used to determine general toxicity profiles for new pharmacological therapies.

## 2. Results

### 2.1. Dendrimer Characterization

**Figure 3:**
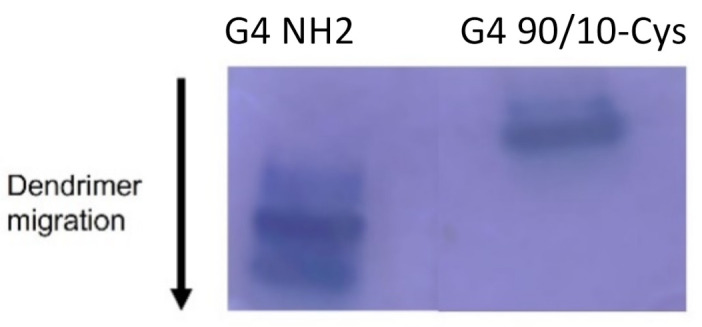
Migration of G4 NH_2_ and G4 90/10-Cys in PAGE gel. From the migration pattern, the dendrimer with 100% amine migrated farthest (lane 1), followed by the dendrimer with 10% amines (lane 2). The migration pattern under acidic conditions reflects the concentration of amines on the dendrimer surface.

Acidic PAGE (Figure 3) was performed to confirm differences in the surface charge composition of G4 90/10-Cys (lane 2) compared to G4 NH_2_ (lane 1). Under these conditions, the dendrimers migrate based on their charge density. Since the size (molecular weight) of G4 90/10-Cys and G4 NH_2_ are quite similar (~14,300 kDa), their migration towards the cathode depends on their surface charge. As expected, G4 NH_2_, which has 100% amines, migrates much faster than the G4 90/10-Cys with 10% surface amines.

**Figure 4:**
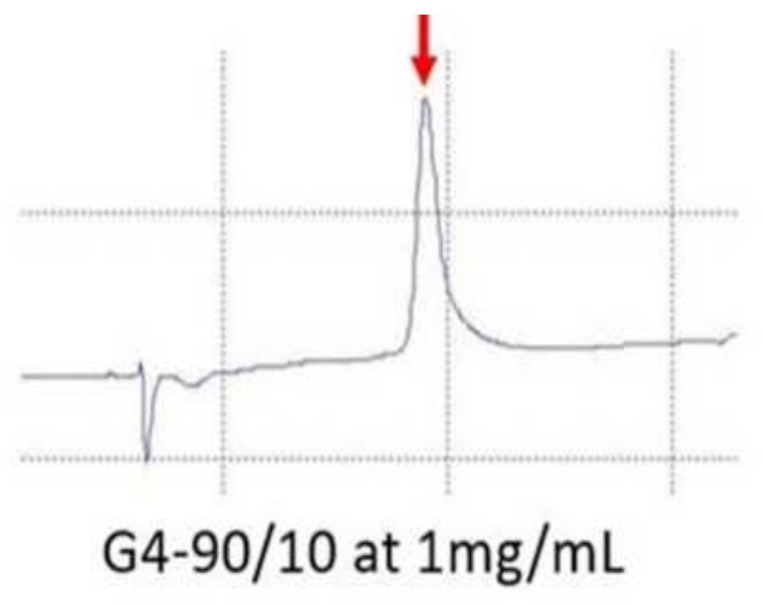
RP-HPLC of G4 90/10-Cys. The peak (red arrow) observed during RP-HPLC confirms that the synthetized dendrimer is pure and homogeneous.

Reverse phase high performance liquid chromatography (RP-HPLC) of G4 90/10-Cys shows that it is a relatively pure product (Figure 4), with a retention time of 10.1 min [25]. 

### 2.2. Reduction of G4 90/10-Cys Dendrimers in the Presence of Glutathione

**Figure 5:**
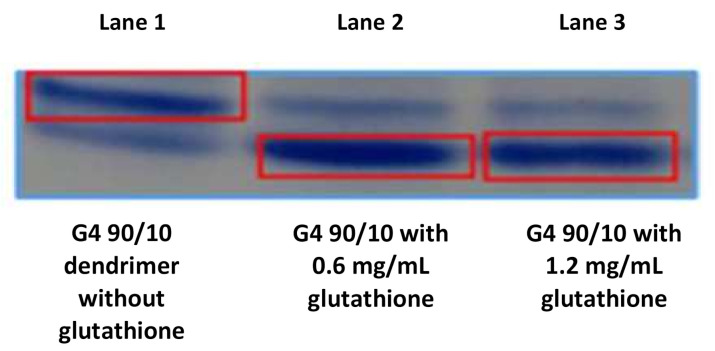
Splitting of G4 90/10 in the presence of glutathione (GSH): Splitting of cystamine dendrimer in the presence of glutathione (GSH): Acid page electrophoresis: Lane 1 contains purified dendrimer; lane 2 contains dendrimer + GSH (0.6 mg/mL); and lane 3 contains dendrimer + GSH (1.2 mg/mL). The top band represents the intact dendrimer while the bottom band represents the reduced dendrimer. The presence of a small amount of reduced dendrimer in lane one is likely the result of reduction during storage. Lanes 2 and 3 show the reduction of the dendrimer in the presence of GSH. It can be clearly seen from lanes 2 and 3 (bottom right band) that glutathione effectively reduces the dendrimer under these conditions.

To confirm that the G4 90/10-Cys dendrimers were reduced by glutathione (0.6 mg/mL and 1.2 mg/mL) compared to a control, the splitting of the dendrimer was assessed. As shown in Figure 5, the dendrimers split in the presence of glutathione (Figure 5). The bottom dark band (that migrates faster towards the cathode—Figure 5 in lane 2 and 3) represents the dendrimer (dendrons) after the parent dendrimer is treated with 0.6 mg/mL glutathione (30 min incubation at room temperature). The faint band that co-migrates with the dendron observed in lane 1 may be the result of natural reduction after synthesis.

### 2.3. Curcumin Release from G4 90/10-Cys

**Figure 6:**
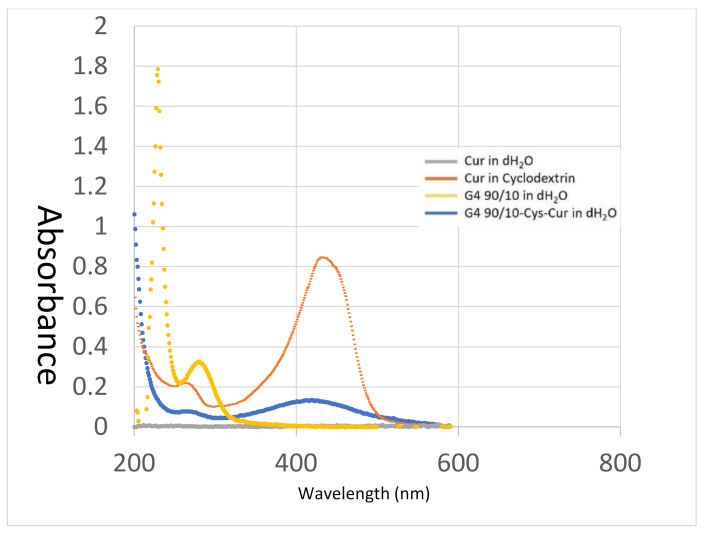
UV-Visible Spectroscopy of encapsulated curcumin. Absorbance spectra of Cur in water (~1 µg/mL—grey line), Cur in cyclodextrin (orange line), G4 90/10-Cys in water (10 mg/mL—yellow line) and G4 90/10-Cys-Cur complexes weight ratio (*w*/*w*) of D to Cur at ~10:1 in distilled water (blue line).

UV-visible spectra of the dendrimer and encapsulated curcumin are shown in Figure 6. Curcumin in water (grey line) shows a very small peak at around 420 nm (barely visible on Figure 6) due to its extremely poor aqueous solubility. Cyclodextrins are cyclic carbohydrates that are also often used to solubilize poorly soluble drugs and other chemicals in water. Curcumin encapsulated in 2-hydroxylpropyl cyclodextrin (orange line) shows a peak at 430 nm. The peak shifted to around 420 nm when curcumin was encapsulated within the dendrimer (blue line). The dendrimer G4 90/10-Cys (yellow line) has a peak at 280 nm but does not show any absorbance above 330 nm. 

**Figure 7:**
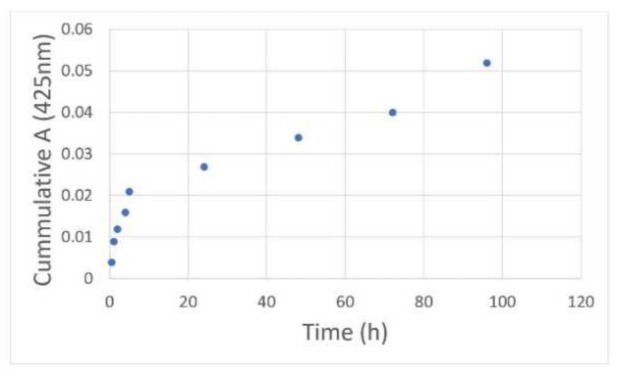
Release kinetics of the encapsulated curcumin: G4 90/10-Cys-Cur was placed in a dialysis tubing (3.5 kDa) and dialyzed against methanol at a constant speed. Samples of the dialysate were taken at various time intervals and their absorbance values at 425 nm were obtained. After removal of each sample of dialysate, fresh PBS was added to maintain the original volume.

The release kinetics of curcumin encapsulated in G4 90/10-Cys is shown in Figure 7. Rapid release was observed in PBS at room temperature over a 4-h period followed by a slower release over the next few days. The initial release (zero-order kinetics; slope = 0.0033; R^2^ = 0.95) may be due to curcumin that is weakly bound to the dendrimer surface or encapsulated within the superficial cavities of the nano-molecule. The second slow-release phase (zero-order kinetics; slope = 0.0003; R^2^ = 0.97) is likely to be from the curcumin encapsulated within the deeper cavities of the G4 90/10-Cys dendrimer.

### 2.4. Effects of G4 90/10-Cys and G4 90/10-Cys-Cur on Different Glioblastoma Cell Lines

Three curcumin-encapsulated dendrimer formulations were tested on various glioblastoma (U87, F98, GL261) and control (HEK 293, Rat MSC, Mouse MSC) cells. The formulations are shown in Table 1.

Curcumin (made from stock dilutions dissolved in DMSO) did not show any effect on the viability of any cell line tested. At the highest dose of curcumin, (0.1 mg/mL), there was 100% cell viability after 24 h incubation of all cell lines (U87, GL261, F98 and non-cancerous cells). In all our results, we compared the viability of cell lines treated with different formulations (Table 1) against untreated cell lines.

#### 2.4.1. Effect of Curcumin Conjugated Dendrimers on Human-Derived Cancer Cells (U87) and Human Kidney Control Cells (HEK 293)

For HEK 293: G4 90/10-Cys did not affect cell viability at any dose; D = 0.02 mg/mL, *p* = 0.68, D = 0.06 mg/mL, *p* = 0.46. D = 1.0 mg/mL *p* = 0.54. For G4 90/10-Cys-Cur, cell viability was lost at the highest dose; D = 0.02 mg/mL, *p* = 0.0151, D = 0.06 mg/mL, *p* = 0.12, D = 1.0 mg/mL, *p* = 0.02. For the cell treated with NH_2_, cell viability was significantly lost at every dose; *p* = 0.0001. 

**Figure 8:**
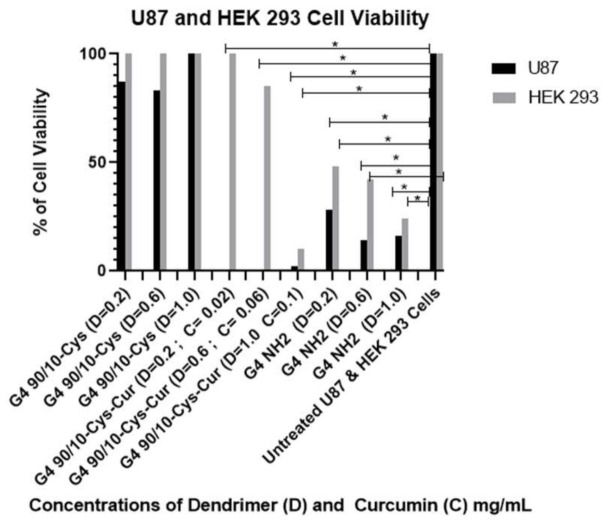
Viability of U87 and non-cancerous HEK 293 control cell after 24 h treatment with G4 90/10-Cys, G4 90/10-Cys-Cur, and G4 NH_2_. G4 90/10-Cys had no significant effect on reducing cell viability, but in fact increased viability at the two higher doses. For U87: G4 90/10-Cys; D = 0.02 mg/mL, *p* = 0.97, D = 0.06 mg/mL, *p* = 0.0009. D = 1.0 mg/mL *p* = 0.0001. For G4 90/10-Cys-Cur *p* < 0.0001 for all three doses. For the cell treated with NH_2_, cell viability was significantly lost at every dose; D = 0.02 mg/mL, *p* = 0.0038, D = 0.06 mg/mL, *p* = 0.0001, D = 1.0 mg/mL, *p* = 0.0001. * *p* < 0.05.

As shown in Figure 8 and Table 2 and Table 3, all formulations of encapsulated curcumin, G4 90/10-Cys-Cur dendrimers (F1–F3), reduced viability of the cancerous cells (U87) compared to Curcumin alone. It is interesting to note that the F3 formulation was only toxic to HEK 293. G4 90/10-Cys by itself showed negligible effects on either the cancer cells or healthy cells, while G4 NH_2_ showed significant killing of both cancer and normal cells.

#### 2.4.2. Effect of Curcumin Conjugated Dendrimers on Rat-Derived Cancer Cells (F98) and Rat Bone Marrow-Derived Mesenchymal Stem Cells (Rat-MSCs)

For Rat-MSCs: G4 90/10-Cys had no significant loss of cell viability at any dose, and significantly increased cell viability at the highest dose. For G4 90/10-Cys; D = 0.02 mg/mL, *p* = 0.68, D = 0.6 mg/mL, *p* = 0.47, D = 1.0 mg/mL *p* = 0.022. For G4 90/10-Cys-Cur, there was no significant loss of cell viability at any dose, and it significantly increased survivability at its lowest and highest doses; D = 0.02 mg/mL *p* = 0.015, D = 0.06 mg/mL, *p* = 0.124, D = 1.0 mg/mL, *p* = 0.022. For G4 NH_2_, *p* < 0.0001 for all three doses.

**Figure 9:**
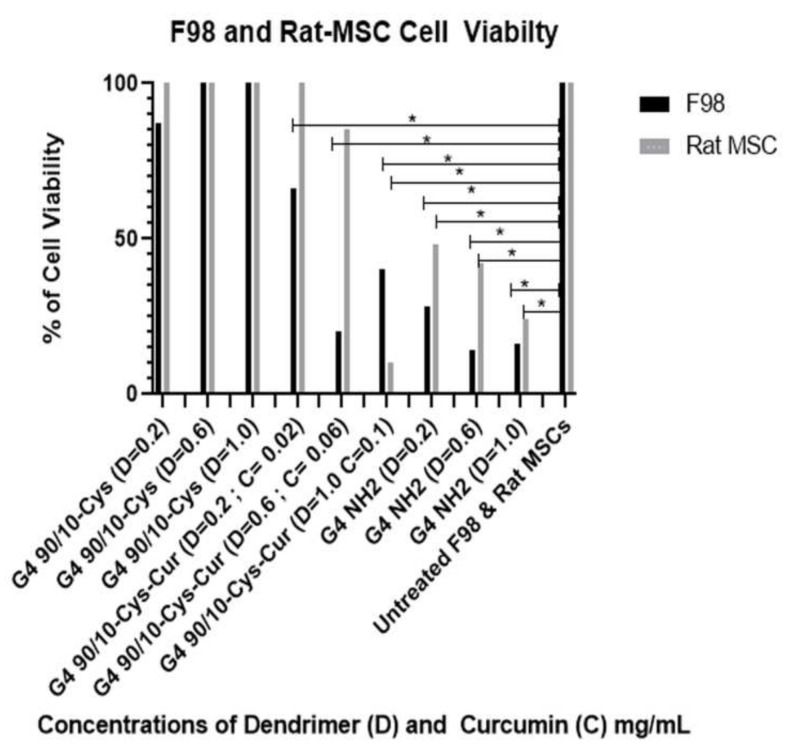
Viability of F98 and non-cancerous rat MSCs after 24 h treatment with G4 90/10-Cys, G4 90/10-Cys-Cur, and G4 NH2. For F98: G4 90/10-Cys there was no loss of cell viability and increased cell viability at the dose of 1.0 mg/mL. For G4 90/10-Cys; D = 0.02 mg/mL, *p* = 0.94, D = 0.47, *p* = 0.017. D = 1.0 mg/mL *p* = 0.0001. For G4 90/10-Cys-Cur *p* < 0.0001 for all three doses. For the cell treated with NH_2_, cell viability was significantly lost at every dose; D = 0.02 mg/mL, *p* = 0.0019, D = 0.06 mg/mL, *p* = 0.0001, D = 1.0 mg/mL, *p* = 0.0001. * *p* < 0.05.

The results shown in Figure 9 and Table 4 and Table 5 demonstrate that curcumin encapsulated G4 90/10-Cys-Cur dendrimers (F1–F3) significantly reduced the viability of F98 cells compared to Curcumin alone. G4 90/10-Cys by itself showed negligible effects on either the cancer cells or healthy cells, while G4 NH_2_ caused significant reduction in the viability of both cancer and normal cells.

#### 2.4.3. Effect of Curcumin Conjugated Dendrimers on Mouse-Derived Cancer Cells (GL261) and Mouse Bone Marrow-Derived Mesenchymal Stem Cells (Mouse-MSCs)

For Mouse MSCs: cell viability was not lost at any dose, and was significantly increased at the higher two doses: G4 90/10-Cys; D = 0.02 mg/mL, *p* = 0.974, D = 0.06 mg/mL, *p* = 0.0009, D = 1.0 mg/mL *p* = 0.0001. For G4 90/10-Cys-Cur *p* < 0.0001 for all three doses. For the control NH_2_, cell viability was significantly lost at every dose; D = 0.02 mg/mL, *p* = 0.0038, D = 0.06 mg/mL, *p* = 0.0001, D = 1.0 mg/mL, *p* = 0.0001.

**Figure 10:**
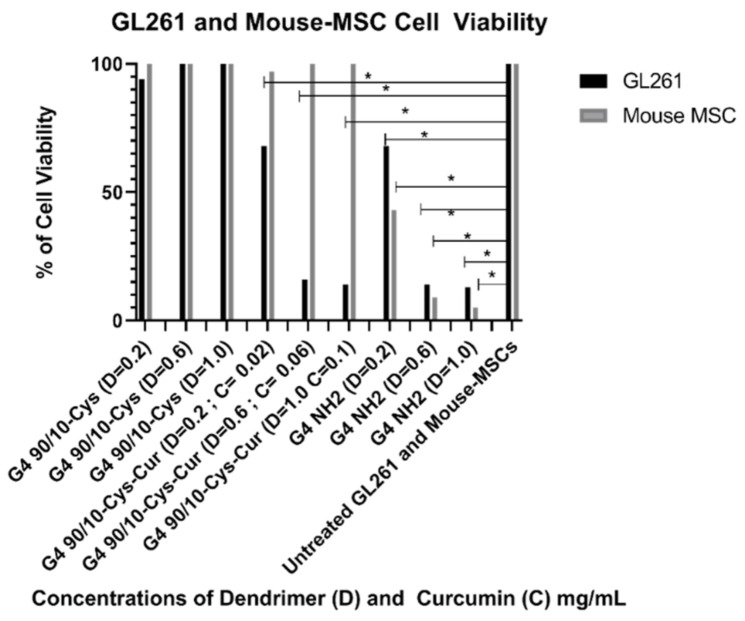
Viability of GL261 and non-cancerous mouse MSCs after 24 h treatment with G4 90/10-Cys, G4 90/10-Cys-Cur, and G4 NH_2_. For GL261, cell viability was not lost at any dose, and was significantly increased at the higher two doses: G4 90/10-Cys; D = 0.02 mg/mL, *p* = 0.974, D = 0.06 mg/mL, *p* = 0.0009, D = 1.0 mg/mL *p* = 0.0001. For G4 90/10-Cys-Cur *p* < 0.0001 for all three doses. For the cell treated with NH_2_, cell viability was significantly lost at every dose; D = 0.02 mg/mL, *p* = 0.0038, D = 0.06 mg/mL, *p* = 0.0001, D = 1.0 mg/mL, *p* = 0.0001. * *p* < 0.05.

Figure 10 and Table 6 and Table 7 demonstrate that curcumin encapsulated G4 90/10-Cys dendrimers (F1–F3) significantly reduced the viability of GL261 cells compared to Curcumin alone. Curcumin by itself, (0.1 mg/mL) did not kill the GL261 cells or control MSCs (100% cell viability; data not shown). G4 90/10-Cys by itself showed negligible effects on either the cancer cells or healthy cells, while G4 NH_2_ significantly reduced the viability of both cancer and normal cells.

## 3. Discussion

Dendrimer nano-molecules are increasingly used in nanotherapeutic research, particularly for the treatment of cancers, due to their small size and ability to encapsulate and deliver non-soluble treatments deep into the tumor. Over the past few years, our lab has focused on the delivery of the polyphenol curcumin, and has found that, by encapsulating this nutraceutical inside fourth generation PAMAM dendrimers, we were able to encapsulate the curcumin, thereby increasing curcumin bioavailability. 

Under our experimental conditions, curcumin by itself was ineffective at killing any of the cancer cell lines (human, rat or mouse). This may be related to its extremely poor solubility in water, thereby decreasing its bioavailability. Encapsulating curcumin into the mixed surface dendrimer (G4 90/10-Cys) showed a remarkable increase in its anti-cancer properties. In the case of the human cancer cell line (U87), even the lowest dose tested (0.02 mg/mL) killed 100% of the cancer cells while showing no effect on the healthy HEK 293 control cells (0% killing). A similar trend was noted with the mid-dose of 0.06 mg/mL encapsulated curcumin. However, at the highest dose (0.1 mg/mL), 90% of healthy HEK 293 cells were also killed. These results suggest that there is an optimum dose range of encapsulated curcumin that would be effective for glioblastoma treatment (Figure 8).

Encapsulated curcumin was less effective in killing mouse (GL 261) or rat (F98) cancer cells. In the case of mouse cells, the lowest dose (0.02 mg/mL) showed about 30% killing while the highest dose (0.1 mg/mL) gave about 80% killing of the cancer cells. None of the doses tested showed any deleterious effects on the control rat or mouse (healthy) cells (Figure 9 and Figure 10).

Concordant with previous research, G4 NH_2_ was found to be extremely toxic, reducing cell viability in all 6 cell types, even at low doses. However, these experiments did support our hypothesis that by reducing the number of surface amines, we reduced dendrimer toxicity to both healthy and cancerous cells. The mixed surface G4 90/10-Cys showed no significant reduction in cell viability in all 3 of the cancer lines, or any of the control lines. We conclude that mixed surface dendrimers are safe and effective candidates for vehicle delivery systems, due to their small (~4 nm) size and ability to encapsulate cargo (curcumin). Additionally, by encapsulating curcumin inside our G4 90/10-Cys we were able to increase the effectiveness of the cargo compared to free curcumin. 

When compared to our G4 NH_2_ toxicity control, we found that our G4 90/10-Cys-Cur was actually more efficient at killing U87 cells than the G4 NH_2_, at all three doses. While this was not reproduced in the rodent cell lines, it shows that the human glioblastoma cell line, in particular, may be more sensitive to treatment with G4 90/10-Cys-Cur. These results will be used to help guide treatment options and concentration doses for a glioblastoma tumor model *in vivo*.

Potential new pharmacotherapeutics emerge from biological sources, chemical molecules, vaccines, and other untapped or re-purposed biological sources. Medicines developed from different sources can respond to genetic predisposition, risk factors, and comorbid conditions, producing potential adverse effects in humans, which makes selection of suitable candidates for clinical trials difficult. This difficulty is mitigated with the help of multi-species drug testing studies [26]. Based on the complexity of using “designer therapies”, it has become a necessary requisite of global regulatory bodies to test new therapeutics in animals prior to human trials (U.S. FDA document: https://www.fda.gov/media/72028/download) (accessed on 25 May 2021). The multispecies preclinical studies provide us with safety and tolerability data of new drugs to support the initial therapeutic dose and long-term drug dosing calculations for clinical trials. Taken together, our treatments with encapsulated curcumin have reduced cellular viability across 3 species while leaving control cells relatively healthy at all but one of those same doses. Additionally, our G4 90/10-Cys and G4 90/10-Cys-Cur have proven to be safe when tested on control tissue at lower doses and, therefore, could be used as an adjunctive treatment or potential vehicle in the treatment of GB.

Temozolomide is the current gold standard of treatment for GB. However, in nearly 50% of patients this drug is ineffective, largely due to a natural drug resistance resulting from overexpression of the MGMT gene. Recent data suggest that curcumin, in addition to its many anticancer effects, may function to prevent drug resistance in certain types of cancer [23]. Other labs have already shown promising results when combining curcumin containing treatments with Temozolomide [27,28]. Treatments with just curcumin have largely failed in clinical trials due to its poor solubility, quick degradation, and elimination from the body before it can deliver its full therapeutic effect. By encapsulating curcumin inside a dendrimer nano-molecule, we increased its therapeutic efficacy by improving bioavailability, its release kinetics over a period of time, reducing the rate at which it is released and broken down and eliminated from the body.

Our findings are limited due to the lack of defined mechanisms underlying the MTT data. Curcumin works on multiple cell cycle pathways and tumor suppressor proteins [29]. It appears that the curcumin is responsible for the reduction in cellular viability of cancer cells, and not the increase in amines on the surface. More investigations are needed to determine if the importance of the curcumin concentration encapsulated in our dendrimers plays a role in the cell viability. However, delineating which specific genes are responsible for these effects is important in tailoring the therapy to individuals or for combined adjunctive treatment. Future *in vitro* studies in this lab will include testing hybrid treatments with G4 90/10-Cys-Cur in addition to a chemotherapeutic, such as Temozolomide, to assess their effects on cancerous and healthy tissue cell lines. Based on the results of the *in vitro* work, future *in vivo* studies will include delivering these treatments into rodent models of glioblastoma to assess their potential where other treatments have failed.

## 4. Materials and Methods

### 4.1. Synthesis of G4 90/10-Cys and Curcumin Encapsulation

G4 90/10-Cys: Surface-modified G4 90/10-Cys dendrimers were synthesized as previously described [25]. Encapsulation was performed by adding G4 90/10 and curcumin at 10:1 (*w*/*w*) ratio in ethanol. After mixing, the ethanol was evaporated in a vacuum oven. The resulting residue was stored in the refrigerator. On the day of the experiment, the residue was reconstituted with Hank’s balanced salt solution (HBSS). The reconstituted formulation was used within a day. The pH of the formulations was around 7. 

### 4.2. Acidic PAGE and RP-HPLC

This was carried out as previously described [30]. Briefly, a 10% acidic PAGE gel was used. Samples were mixed with 50% sucrose and added to the wells. Coomassie blue was used to stain the gel. For HPLC, a C18 column was used with Hitachi HPLC system (Hitachi, Tokyo, Japan). Mobile phase A was water (with 0.1% trifluoroacetic acid) and mobile phase B was 0.085% trifluoroacetic acid in acetonitrile.

### 4.3. Animals

Donor for the mouse cells were 1 male and 2 female C57BL/6J mice, bred in-house aged between 10 and 15 weeks old. All mice were group-housed in a clear polycarbonate container with sawdust bedding and access to food and water ad libitum. The room temperature was set to 22 °C and on the room is under control of an automatic timer creating a 12 h light/dark cycle (lights ON at 12:00 a.m.). The experimental procedures are approved by IACUC of CMU (CMU IACUC protocol #18−23).

Donors for the rat cells were 1 male and 2 female Sprague Dawley rats, bred in-house aged between 10–15 weeks old. All rats were group-housed in a clear polycarbonate container with sawdust bedding and access to food and water *ad libitum*. The room temperature was set to 22 °C and on the room is under control of an automatic timer creating a 12 h light/dark cycle (lights ON at 12:00 a.m.). The experimental procedures are approved by IACUC of CMU (CMU IACUC protocol #18−12).

### 4.4. Maintenance of Different Cell Lines

#### 4.4.1. U87

Human glioblastoma U87 cells: These cells were purchased from ATCC (American Type Tissue Collection, Manassas, VA, USA). Cells were cultured in Dulbecco’s Modified Eagle Medium (DMEM; Gibco, Waltham, MA, USA), with 10% Fetal Bovine Serum (FBS; Fisher Scientific, Waltham, MA, USA), and 1% penicillin/Streptomycin (Gibco, Waltham, MA, USA) in T-75 flasks and incubated at 37 °C with 5% CO_2_. Cells were cultured until 80% confluent. The media was removed, and the cells were washed with PBS, and 3 mL of 0.25% trypsin (Gibco, Waltham, MA, USA) was added to the flask and dispersed evenly until cells began to dissociate at 37 °C for 3–5 min. The trypsin was removed and the cells were placed in a 15 mL conical tube with 3 mL of fresh media, which was centrifuged at 1200 rpm for 7 min. The supernatant was removed, leaving a pellet of cells at the bottom of the tube. One mL of fresh media was added and mixed gently. An aliquot of 10 µL of this cell/media mixture was then used to count cells using a hemocytometer, after which the cells were re-plated in a 96 well plate at a density of 10,000 cells per well. A stock of cells was cultured until passage 7 and then stored in a −80 °C ultra-freezer until further use. All cells used in this study were passaged the same way and used at passage 8 to eliminate variability.

#### 4.4.2. HEK 293

Human Embryonic Kidney 293 cells: Cells were purchased from American Type Culture Collection (ATCC, Manassas, VA, USA). Cells were cultured as previously described in DMEM (Gibco, Waltham, MA, USA), with 10% FBS (Fisher Scientific, Waltham MA, USA), and 1% penicillin/streptomycin (PS; Gibco, Waltham, MA, USA) in T75 cm^2^ (T-75) tissue culture flasks and incubated at 37 °C at 5% CO_2_, until 75% confluent. They were then passaged and re-plated in a 96 well plate at 10,000 cells per well for use.

#### 4.4.3. F98

Rat cancer cells were purchased from ATCC (American Type Tissue Collection, Manassas, VA, USA). Cells were cultured in DMEM (Gibco, Waltham, MA, USA) with 10% FBS (Fisher Scientific, Waltham, MA, USA) and 1% PS in T-75 and incubated at 37 °C with 5% CO_2_ until 75% confluent. Cells were then passaged and replated in a 96 well plate at 10,000 cells per well until use.

#### 4.4.4. Rat MSCs

Rat bone marrow-derived MSCs were isolated and cultured, as previously described [30]. In brief, 3 adult Sprague Dawley rats were euthanized under anesthesia, and the femur and tibia of both sides were removed. After washing with sterile PBS, the bones were flushed out with α-MEM (Gibco, Waltham, MA, USA) to get bone marrow cells. Cells were then plated in a T-25 in α-MEM with 10% FBS (Gibco, Waltham, MA, USA), 10% horse serum (HS; Gibco, Waltham, MA, USA), and 1% PS (Gibco, Waltham, MA, USA).

#### 4.4.5. GL261 Red-FLuc

Mouse glioma cells were purchased from Bioware Brite (BW134246V), Perkin Elmer (Waltham, MA, USA) pre-engineered with a firefly luciferase gene from Luciola Italica (Red-FLuc, Waltham, MA, USA). Cells were cultured in Dulbecco’s Modified Eagle Medium (DMEM; Gibco, Waltham, MA, USA), with 10% Fetal Bovine Serum (FBS; Fisher Scientific, Waltham, MA, USA), and 1% PS (Gibco, Waltham, MA, USA) in T-75 flasks and incubated at 37 °C with 5% CO_2_. Cells were cultured until 75% confluent. Cells were then passaged and replated in a 96 well plate at 10,000 cells per well until use.

#### 4.4.6. Mouse MSCs

Mouse bone marrow-derived MSCs were isolated and cultured, as previously described [31]. In brief, 3 adult C57BL/6J mice were euthanized and the femur and tibias from each side were taken. After washing in sterile PBS, the bones were flushed with DMEM (Gibco, Waltham, MA, USA). Cells were then plated in a T-25 in DMEM with 10% FBS (Gibco, Waltham, MA, USA), 10% HS (Gibco, Waltham, MA, USA), and 1% PS (Gibco, Waltham, MA, USA).

### 4.5. MTT Assay

**Figure 11:**
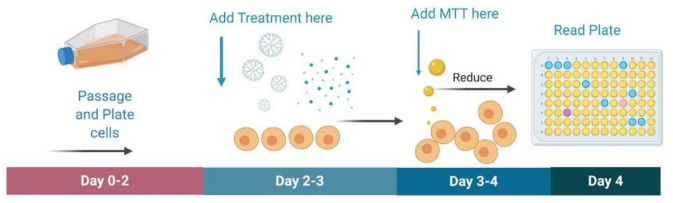
Schematic of MTT assay. For the MTT assay, cells are passaged and plated in a 96 well plate. Once confluent, treatment is added in triplicates to incubate for 24 h. Afterwards, MTT dye is added and incubated for four hours. MTT detergent reagent is then added and incubated for 2 h in the dark. Plates are then read on a spectrophotometer at 570 nm.

U87 and their control HEK 293; F98 and their control Rat MSC; GL261, and their control mouse MSCs were each plated in 96 well plates for MTT assay using the protocol previously established by ATCC (American Type Culture Collection, Manassas, VA, USA; Figure 11). Unencapsulated curcumin, G4 NH_2_, G4 90/10-Cys, and G4 90/10-Cys-Cur dendrimers were tested in concentrations of 0.2, 0.6, 1.0 mg/mL on all cell lines. In brief, 10,000 cells were plated in each well in 100 μL of media. Once the cells were 70% confluent, the treatments were added in triplicates at each concentration, leaving 3 wells for untreated cells and 3 wells with just media as an internal control. Twenty-four hours following treatment, the media was removed and 100 μL of fresh media was added to each well. The MTT reagent was dissolved in deionized water at a concentration of 5 mg/mL, and 10 μL was added to each well and then placed back in the incubator for four hours at 37 °C with 5% CO_2_. The plates were then removed from the incubator and 100 μL of detergent reagent consisting of 0.01 M hydrochloric acid in 10% sodium dodecyl sulfate was added to each well. The plates were then covered and left at room temperature for two hours in the dark and were then read on a spectrophotometer at an absorbance of 570 nm. Optimal doses can be assessed by determining which causes maximal cell death in the glioblastoma cells without affecting the control cells. During the MTT assay, all treatments and controls were performed in triplicate. Percent cell viability was determined by first averaging the absorbance rates of the three untreated wells of cells. Their average absorbance was then used as a control set at 100% cell viability. The absorbance rate triplicates for each sample were averaged and divided by the control to obtain the percentage of cell viability for each treatment.

### 4.6. Statistical Analysis

All statistical analysis was done using GraphPad Prism 8 (GraphPad, San Diego, CA, USA). One-way ANOVA with Tukey’s Post Hoc comparison was done to assess significant reductions in cell viability using an MTT assay. An alpha level of *p* < 0.05 was used for all analyses.

## 5. Conclusions

A formidable barrier to treating GB is delivering therapeutics to the tumor in a safe and efficient way. The novel PAMAM dendrimer described in this study represents a first step as a promising method of overcoming this major obstacle. Our study demonstrated that delivering curcumin (which otherwise has limited bioavailability) to cancer cells *in vitro* is lethal to different glioblastoma cell lines from three different species. Our new method of encapsulation of curcumin seems to be only toxic to cancer cells and therefore could be a new alternative to treat the devastating glioblastoma.

## Figures and Tables

**Table 1:** G4 90/10-Cys encapsulated with varying amounts of curcumin.

Formulation	[G4 90/10-Cys], mg/mL	[Curcumin], mg/mL
F1	0.2	0.02
F2	0.6	0.06
F3	1.0	0.1

**Table 2:** Effect of F1, F2 and F3 on U87 cells.

Formulation	Dendrimer	[Dendrimer] mg/mL	[Curcumin] mg/mL	Cell Viability (%)	*p* Value
F1	G4 90/10-Cys	0.2	0.02	0	<0.0001
F2	G4 90/10-Cys	0.6	0.06	0	<0.0001
F3	G4 90/10-Cys	1	0.1	2	<0.0001
Dendrimer control	G4 90/10-Cys	0.2	0	87	0.97
Dendrimer control	G4 90/10-Cys	0.6	0	83	0.0009
Dendrimer control	G4 90/10-Cys	1	0	100	<0.0001
Dendrimer control	G4 NH_2_	0.2	0	28	0.0038
Dendrimer control	G4 NH_2_	0.6	0	14	<0.0001
Dendrimer control	G4 NH_2_	1	0	16	<0.0001

**Table 3:** Effect of F1, F2 and F3 on control HEK 293 cells.

Formulation	Dendrimer	[Dendrimer] mg/mL	[Curcumin] mg/mL	Cell Viability (%)	*p* Value
F1	G4 90/10-Cys	0.2	0.02	100	0.0151
F2	G4 90/10-Cys	0.6	0.06	85	0.12
F3	G4 90/10-Cys	1	0.1	10	0.02
Dendrimer control	G4 90/10-Cys	0.2	0	100	0.68
Dendrimer control	G4 90/10-Cys	0.6	0	100	0.46
Dendrimer control	G4 90/10-Cys	1	0	100	0.54
Dendrimer control	G4 NH_2_	0.2	0	48	<0.0001
Dendrimer control	G4 NH_2_	0.6	0	42	<0.0001
Dendrimer control	G4 NH_2_	1	0	24	<0.0001

**Table 4:** Effect of F1, F2 and F3 and on F98 cells.

Formulation	Dendrimer	[Dendrimer] mg/mL	[Curcumin] mg/mL	Cell Viability (%)	*p* Value
F1	G4 90/10-Cys	0.2	0.02	66	0.0015
F2	G4 90/10-Cys	0.6	0.06	20	<0.0001
F3	G4 90/10-Cys	1	0.1	40	<0.0001
Dendrimer control	G4 90/10-Cys	0.02	0	85	0.94
Dendrimer control	G4 90/10-Cys	0.06	0	100	0.47
Dendrimer control	G4 90/10-Cys	0.1	0	100	0.017
Dendrimer control	G4 NH_2_	0.2	0	53	0.0019
Dendrimer control	G4 NH_2_	0.6	0	19	<0.0001
Dendrimer control	G4 NH_2_	1	0	21	<0.0001

**Table 5:** Effect of F1, F2 and F3on Rat MSC control cells.

Formulation	Dendrimer	[Dendrimer] mg/mL	[Curcumin] mg/mL	Cell Viability (%)	*p* Value
F1	G4 90/10-Cys	0.2	0.02	100	0.015
F2	G4 90/10-Cys	0.6	0.06	100	0.124
F3	G4 90/10-Cys	1	0.1	100	0.022
Dendrimer control	G4 90/10-Cys	0.02	0	100	0.68
Dendrimer control	G4 90/10-Cys	0.06	0	100	0.47
Dendrimer control	G4 90/10-Cys	0.1	0	100	0.18
Dendrimer control	G4 NH_2_	0.2	0	38	<0.0001
Dendrimer control	G4 NH_2_	0.6	0	0	<0.0001
Dendrimer control	G4 NH_2_	1	0	2	<0.0001

**Table 6:** Effect of F1, F2 and F3 on GL261 glioblastoma cells.

Formulation	Dendrimer	[Dendrimer] mg/mL	[Curcumin] mg/mL	Cell Viability (%)	*p* Value
F1	G4 90/10-Cys	0.2	0.02	68	<0.0001
F2	G4 90/10-Cys	0.6	0.06	17	<0.0001
F3	G4 90/10-Cys	1	0.1	22	<0.0001
Dendrimer control	G4 90/10-Cys	0.02	0	94	0.974
Dendrimer control	G4 90/10-Cys	0.06	0	100	0.0009
Dendrimer control	G4 90/10-Cys	0.1	0	100	<0.0001
Dendrimer control	G4 NH_2_	0.2	0	68	0.0038
Dendrimer control	G4 NH_2_	0.6	0	14	<0.0001
Dendrimer control	G4 NH_2_	1	0	13	<0.0001

**Table 7:** Effect of F1, F2 and F3 on Mouse MSC control cells.

Formulation	Dendrimer	[Dendrimer] mg/mL	[Curcumin] mg/mL	Cell Viability (%)	*p* Value
F1	G4 90/10-Cys	0.2	0.02	97	>0.999
F2	G4 90/10-Cys	0.6	0.06	100	0.38
F3	G4 90/10-Cys	1	0.1	100	0.93
Dendrimer control	G4 90/10-Cys	0.02	0	100	>0.999
Dendrimer control	G4 90/10-Cys	0.06	0	100	0.98
Dendrimer control	G4 90/10-Cys	0.1	0	100	>0.99
Dendrimer control	G4 NH_2_	0.2	0	43	0.026
Dendrimer control	G4 NH_2_	0.6	0	0	<0.0001
Dendrimer control	G4 NH_2_	1	0	0	0.0002

## Data Availability

Not applicable.

## Sample Availability

Samples of the dendrimers and the dendrimer encapsulated curcumin are available from the corresponding author.

## Abbreviations

ATCC	American Type Tissue Collection
G4 90/10	Generation 4, 90% hydroxyl, 10% amines
G4 90/10-Cys	Generation 4, 90% hydroxyl, 10% amines, cystamine core
G4 90/10-Cys-Cur	Generation 4, 90% hydroxyl, 10% amines, cystamine core with curcumin
BBB	Blood Brain Barrier
BBTB	Blood Brain Tumor Barrier
Cur	Curcumin
Cys	Cystamine
DMEM	Dulbecco’s Modified Eagle Medium
FBS	Fetal Bovine Serum
GB	Glioblastoma
GSH	Glutathione
MGMT	O^6^-methylguanine methyltransferase
MTT	3-(4,5-dimethylthiazol-2-yl)-2,5-diphenyltetrazolium bromide
NH_2_, NH_3_^+^	Amines
OH	Hydroxyl
PAGE	Polyacrylamide gel electrophoresis
PAMAM	Polyamidoamine
PBS	Phosphate Buffer Saline
RP-HPLC	Reverse Phase High-Performance Liquid Chromatography
TMZ	Temozolomide
MSC	Mesenchymal stem cells
HEK	Human embryonic Kidney
HS	Horse serum
PS	Penicillin Steptomycin
